# Genetic complexity and multiple infections with more Parvovirus species in naturally infected cats

**DOI:** 10.1186/1297-9716-42-43

**Published:** 2011-03-02

**Authors:** Mara Battilani, Andrea Balboni, Martina Ustulin, Massimo Giunti, Alessandra Scagliarini, Santino Prosperi

**Affiliations:** 1 Department of Veterinary Medical Sciences, Alma Mater Studiorum, University of Bologna, Italy

## Abstract

Parvoviruses of carnivores include three closely related autonomous parvoviruses: canine parvovirus (CPV), feline panleukopenia virus (FPV) and mink enteritis virus (MEV). These viruses cause a variety of serious diseases, especially in young patients, since they have a remarkable predilection for replication in rapidly dividing cells. FPV is not the only parvovirus species which infects cats; in addition to MEV, the new variants of canine parvovirus, CPV-2a, 2b and 2c have also penetrated the feline host-range, and they are able to infect and replicate in cats, causing diseases indistinguishable from feline panleukopenia. Furthermore, as cats are susceptible to both CPV-2 and FPV viruses, superinfection and co-infection with multiple parvovirus strains may occur, potentially facilitating recombination and high genetic heterogeneity. In the light of the importance of cats as a potential source of genetic diversity for parvoviruses and, since feline panleukopenia virus has re-emerged as a major cause of mortality in felines, the present study has explored the molecular characteristics of parvovirus strains circulating in cat populations. The most significant findings reported in this study were (a) the detection of mixed infection FPV/CPV with the presence of one parvovirus variant which is a true intermediate between FPV/CPV and (b) the quasispecies cloud size of one CPV sample variant 2c. In conclusion, this study provides new important results about the evolutionary dynamics of CPV infections in cats, showing that CPV has presumably started a new process of readaptation in feline hosts.

## Background

Parvoviruses are non-enveloped single-stranded DNA viruses which infect a wide range of mammalian species, including several members of the order Carnivora. Parvoviruses of carnivores include three closely related autonomous parvoviruses: canine parvovirus (CPV), feline panleukopenia virus (FPV) and mink enteritis virus (MEV). These viruses cause a variety of serious diseases, especially in young patients, since they have a remarkable predilection for replication in rapidly dividing cells, such as bone marrow, enteric epithelium and the foetus. Feline panleukopenia virus (FPV), the prototype parvovirus of carnivores, is responsible for a homonymous disease, a highly contagious sickness occurring in cats, characterized by severe leukopenia, gastroenteritis, reproductive disorders and nervous signs [1].

FPV is not the only parvovirus species affecting cats; in addition to MEV, the new variants of canine parvovirus, CPV-2a, 2b [2] and 2c [3] have also penetrated the feline host-range, and they are able to infect and replicate in cats, causing diseases indistinguishable from feline panleukopenia.

In consequence of the host range shift of CPV-2 from dog to cats, natural infections of cats and wild felines with CPV-2 have been reported [4], but FPV remains the more prevalent species of parvoviruses causing disease in cats [5].

Recent investigations carried out by characterization at the molecular level of the parvovirus strains detected in cats with gastroenteritis have also shown a clear prevalence of FPV over CPV-2 in naturally infected cats [3,6]. On the contrary, in some Asian countries, more than 80% of the isolates from cats and wild felines were CPV types, but the precise mechanism of the predominance of CPV-2 in cats in these areas remains obscure [7].

Furthermore, since cats are susceptible to both CPV-2 and FPV viruses, superinfection and co-infection with multiple parvovirus strains may occur, potentially facilitating recombination and high genetic heterogeneity [8-11].

In the light of the importance of cats as a potential source of genetic diversity for parvoviruses and, since feline panleukopenia virus has re-emerged as a major cause of mortality in felines, parvovirus strains detected in cats with clinical signs of panleukopenia were analyzed, and molecular characterization, sequence diversity and genetic complexity were evaluated on the VP2 capsid gene.

The detection of an FPV/CPV mixed infection in one infected cat with the presence of a parvovirus variant which is a true intermediate between CPV and FPV and the quasispecies cloud size of one CPV sample variant 2c were the most significant findings reported in this study.

## Materials and methods

### Samples

The sample population included 24 cats, under 15 months of age, with a clinical diagnosis of parvovirus infection and at least one biological specimen (fecal or gut samples) sent to the Laboratory of Virology of the Department of Veterinary Medical Sciences (DVMS - University of Bologna, Italy) for diagnostic purposes between March 2000 and November 2009. The biological samples collected were frozen and stored at -80°C for later analysis. Confirmation of parvovirus infection was carried out by PCR amplification as previously described [12]. The characteristics of the samples examined are summarized in Table 1.

**Table 1:** Summary of biological specimens examined in this study.

Virus	Specimen	Year of collection	Age	Clinical presentation	Vaccination status	Origin
702	Faeces	2000	2M	Gastroenteritis	none	Owner
713	Gut	2001	2M	Gastroenteritis	none	Owner
671	Gut	2002	2M	Gastroenteritis	none	Pet's store
1076	Rectal swab	2002	15M	Gastroenteritis	none	Shelter
1469	Rectal swab	2002	2M	Gastroenteritis	none	Owner
828	Gut	2003	2M	Sudden death	none	Shelter
829	Rectal swab	2003	2A	none	Complete	Shelter
1759	Gut	2003	2M	Gastroenteritis	none	Owner
1306	Gut	2004	2M	Gastroenteritis	none	Shelter
339	Gut	2006	1A	Gastroenteritis	none	Shelter
1897	Rectal swab	2006	nd	Gastroenteritis	none	Owner
159	Rectal swab	2007	9M	Gastroenteritis	Complete	Owner
173	Rectal swab	2007	7M	Gastroenteritis	Complete	Owner
239	Rectal swab	2007	7M	Gastroenteritis	Complete	Owner
398	Rectal swab	2007	3A	Gastroenteritis	Complete	Owner
998	Faeces	2008	3M	Gastroenteritis	none	Shelter
1033	Gut	2009	40D	Sudden death	none	Shelter
1034	Gut	2009	40D	Sudden death	none	Shelter
1035	Gut	2009	40D	none	none	Shelter
1036	Gut	2009	40D	Gastroenteritis	Incomplete	Shelter
1037	Gut	2009	40D	Gastroenteritis	none	Shelter
1038	Gut	2009	3M	Gastroenteritis	Incomplete	Shelter
1039	Gut	2009	40D	Gastroenteritis	none	Shelter
1040	Gut	2009	40D	Gastroenteritis	none	Shelter

Age of the cat at presention: Y = year, M = months, D = days

Feline panleukopenia modified-live vaccines.

In most cases, cats had a history of incomplete or no vaccinations. The clinical symptoms most frequently observed in our population included fever, anorexia, gastro-intestinal signs (diarrhea, vomiting), depression, weakness and leukopenia. In most of the subjects, the clinical course was mild and chronic while two patients (829/03 and 1035/09) were clinically healthy. However, 8 cats included in the study showed a more severe manifestation of the disease: 3 of them (828/03, 1033/09 and 1034/09), actually died suddenly without showing any clinical signs; the other 5 were referred to the Veterinary Teaching Hospital of the University of Bologna (VTH-UB) for a severe and acute manifestation of the disease and died shortly after admission, despite intensive therapy.

Fourteen cats came from feline colonies where recurrent cases of parvovirus infections occurred with a seasonal cadence.

### Molecular characterisation of viral strains

#### Extraction of viral DNA, Polymerase Chain Reaction (PCR) of the VP2 gene and consensus sequencing

Viral DNA was extracted from specimens using a Nucleospin Tissue mini kit (Macherey-Nagel, Düren, Germany) according to the manufacturer's instructions.

The entire VP2 gene was amplified with a set of primers designed for both feline and canine parvovirus (P1 forward 5'-ATGAGTGATGGAGCAGTTC-3' and VP reverse 5'-TTCTAGGTGCTAGTTGAG-3'). Reactions of amplification were carried out using the Klen Taq LA Polymerase Mix (Clontech-TAKARA Bio Company, Mountain View, CA, USA) which contains KlenTaq-1 DNA polymerase as the primary polymerase and a small amount of a 3' → 5' proofreading polymerase for high fidelity PCR. The temperature cycling protocol consisted of 40 cycles of denaturation at 94°C for 30 s, annealing at 54°C for 2 min, and extension at 72°C for 2 min, with a final extension at 72°C for 10 min. A 5 μL aliquot of each PCR product was analyzed by electrophoresis using a 1% agarose gel and ethidium bromide staining.

The targeted DNA fragments were purified using a PCR product purification kit (Roche, Mannheim, Germany) and sequenced in both orientations, sense and antisense, using standard dideoxy sequencing with fluorescence-labelled nucleotides (Applied Biosystem-PerkinElmer, Carlsbad, CA, USA).

Direct sequencing of the PCR products of two viral strains, 1469/02 and 998/08, showed an unusually high number of ambiguities; although a proofreading Taq was used, these results suggested mixed viral populations; therefore, the amplification products were cloned into the PCR 4/TOPO vector using the TOPO cloning kit (Invitrogen, Carlsbad, CA, USA), and were transformed into *Escherichia coli *DH5α-competent cells according to the manufacturer's protocol. Eight and ten recombinant clones for the strains 1469/02 and 998/08, respectively, were purified with Turbo Kit (QBIOgene-MP Biomedicals, Montreal, Canada) and were sequenced.

#### Sequence data

The complete VP2 nucleotide sequences obtained were compared to sequences available from the GenBank and alignment of the sequences was carried out using the CLUSTAL W web interface. The alignments are available from the authors on request.

Translation of the nucleotide sequences into amino acid sequences and the degree of similarity among the sequences at both the nucleotide and the amino acid levels was determined using the BIOEDIT sequence alignment editor version 7.0.9.

A variety of statistical analyses regarding nucleotide diversity and sequence variability were carried out on the sequence data set using the versatile program DNASP version 5.10.00 [13]. Statistical analysis was carried out on the subpopulations, grouping the sequence data on the basis of the parvovirus species and the antigenic types.

The following parameters were considered: the number of polymorphic sites *S*, the nucleotide diversity Pi (*π*) [14] which is the average number of substitutions between any two sequences and it standard deviation (standard error), and the total number of synonymous and non-synonymous differences.

Mutation frequency (total number of changes/total number of bases sequenced) and the percentage of mutated clones were used as indicators of genetic diversity of the viral population of strains 1469/02 and 998/08.

To examine the evolutionary pressures influencing the parvovirus evolution inside the host over time, the ratio of non-synonymous substitutions per non-synonymous site (dN) to synonymous substitutions per synonymous site (dS) were estimated using the Datamonkey web interface, a maximum likelihood-based tool for the identification of sites subject to positive or negative selection. Datamonkey implements three complementary methods: single likelihood ancestor counting (SLAC), fixed effects likelihood (FEL) and random effects likelihood (REL). The HKY85 model was employed for the entire data set and for sample 998/08 whereas, for sample 1469/02, the F81 substitution model was applied.

Since genetic recombination has been assessed as a factor in parvovirus evolution [10,11], and recombination events could affect phylogenetic and pressure analyses, a sequence dataset was screened for recombination using Genetic Algorithms for Recombination Detection (GARD) [15].

Maximum likelihood (ML) phylogenetic trees were estimated using PAUP* version 4.0, with the best-fit model of nucleotide substitution determined using Modeltest [16]. The TrN + I + Γ substitution model was optimal for all the sequence data (including reference strains), whereas the GTR + I + Γ substitution model was used for the sequences analyzed in this study; the key parameter values (the TrN and GTR substitution matrix, the proportion of invariant sites I and the gamma distribution Γ of rate variation with eight categories) were estimated from the data given. To assess support for individual nodes, bootstrap resampling values were estimated with 1000 neighbor-joining trees, again employing PAUP*.

The following reference FPV strains detected in various parts of the world were obtained from GenBank and included in the phylogenetic analysis: Asia (accession numbers: AB000052; AB000054; AB000056; AB000059; AB000061; AB000064; AB000066; AB000068; AB000070; AB054225; AB054226; AB054227; AF015223; D78584; D88286; D88287; DQ474236; DQ474237; EF988660; EU252145; EU252146; EU252147; EU697383; EU697384; EU697386; EU697387; FJ231389; FJ405225; FJ93617); Australia (accession number: X55115); Europe (accession numbers: AY665655; EF418569; DQ474238; EU145593; EU221279; EU221280; EU360958; EU360959; EU498713; EU498714; EU498716; EU498717; EU498719; FPU22188); Italy (accession numbers: EU498682; EU498683; EU498684; EU498685; EU498690; EU468692; EU498693; EU498694; EU498695; EU498697; EU498698; EU498699; EU498700; EU498701; EU498704; EU498705; EU498706; EU498707; EU498708; EU498709; EU498710; EU498711; EU498712; EU498715; EU498720); South America (accession numbers: FJ440712; EU018142; EU018143; EU018144; EU018145; FJ440714); USA (accession numbers: EU659111; EU659112; EU659113; EU659114; EU659115; M10824; M24002; M24004; U22187; U22189).

## Results

In this study we analyzed 24 parvovirus strains detected in domestic cats. Molecular detection of the parvoviruses was carried out on biological specimens by applying PCR, and the VP2 gene sequences were determined.

The nucleotide sequences of the VP2 gene obtained were 1745 bp in length. Analysis of the deduced amino acid residues at critical positions allowed the identification of the parvovirus species: 22 viral strains were FPV while, for two strains, 1469/02 and 998/08, it was necessary to proceed with the cloning of the amplicons, since the direct sequencing of PCR products showed an unusually high number of ambiguites, suggesting the presence of mixed viral populations. The amplification products of 1745 bp of strains 1469/02 and 998/08 were cloned, and 8 and 10 clones respectively, were chosen for sequencing.

Comparison of the nucleotide sequences of the 1469/02 clones evidenced 8 different viral populations with a sequence similarity at the nucleotide level which varied from 99.8 to 99.9%. At the amino acid level, clones 2-6-7 were identical while the remaining clones differed in identity by 99.5 to 99.8%. Analysing the amino acid positions which distinguish the feline and canine parvovirus species and the antigenic types of CPV, it was seen that all clones of 1469/02 belonged to the new variant CPV-2c.

Sequence analysis of 10 clones of strain 998/08 showed complete identity between clone 6 and clone 20 as well as between clones 1, 3, 4 and 7 at the nucleotide level; the other clones showed a sequence similarity which varied from 98.3 to 99.9%. On the basis of the deduced amino acid sequences, clones 1-3-4-7-12-13 were identical; the remaining clones showed a sequence similarity which varied from 97.6 to 99.8%. Analysis of the critical residues which affect the host-range and antigenic properties of parvoviruses of carnivores, showed that clones 6, 15 and 20 were CPV-2a; instead, clones 1, 3, 4, 7, 9, 12, 13 were FPV, with evidence of a mixed infection involving two species of parvovirus in the same patient.

Regarding the 22 FPV strains, comparison of the nucleotide sequences showed a 100% similarity between the following strains: 828/03 and 829/03; 159/07, 239/07 and 398/07; 1033/09, 1034/09, 1035/09, 1036/09, 1037/09, 1038/09, 1039/09 and 1040/09. In the remaining strains, the similarity ranged from 99.1 to 99.8%. At the amino acid level, strains 828/03, 713/01, 239/07, 398/07, 173/07, 1897/06 and clones 1, 3, 4, 7, 12, 13 of strain 998/08, as well as strains 339/06 and 1076/02 were identical; the other strains showed a sequence of similarity which varied from 99.6 to 99.8.

Several synonymous and non-synonymous substitutions were detected by comparing the sequences. The predicted amino acid sequence substitutions are summarized in Table 2. The sequences obtained were compared to parvovirus reference strains retrieved from the GenBank database; the alignments showed that, of the 6 non-synonymous changes, at least 2 were very common since the reference strains also showed them and they are the coding change in residue 13 (Pro→Ser) and residue 91 (Ala→Ser). The VP2 Asn 426-to-Ser, Lys 501-to-Arg, Asn 517-to-Ser changes were unique and characteristic of our strains.

**Table 2:** Change of amino acids in the sequence of VP2 of the viral strains.

Amino acid position																												
**SAMPLES**	**13**	**46**	**80**	**87**	**91**	**93**	**98**	**103**	**110**	**232**	**282**	**297**	**300**	**305**	**323**	**340**	**373**	**415**	**426**	**440**	**482**	**498**	**501**	**511**	**517**	**564**	**568**	**TYPE**

FPV^a^	Pro	Asn	Lys	Met	Ala	Lys	Leu	Val	Ser	Val	Asn	Ser	Ala	Asp	Asp	Ala	Asp	Ile	Asn	Thr	Leu	Leu	Lys	Asp	Asn	Asn	Ala	
CPV-2^b^	-	-	Arg	-	-	Asn	-	Ala	-	Ile	-	-	-	-	Asn	-	-	-	-	-	-	-	-	-	-	Ser	Gly	
CPV-2a^b^	-	-	Arg	Leu	-	Asn	-	Ala	-	Ile	-	-	Gly	Tyr	Asn	-	-	-	-	-	-	-	-	-	-	Ser	Gly	
CPV-2b^b^	-	-	Arg	Leu	-	Asn	-	Ala	-	Ile	-	-	Gly	Tyr	Asn	-	-	-	Asp	-	-	-	-	-	-	Ser	Gly	
CPV-2a^c^	-	-	Arg	Leu	-	Asn	-	Ala	-	Ile	-	Ala	Gly	Tyr	Asn	-	-	-	-	-	-	-	-	-	-	Ser	Gly	
CPV-2b^c^	-	-	Arg	Leu	-	Asn	-	Ala	-	Ile	-	-	Gly	Tyr	Asn	-	-	-	Asp	-	-	-	-	-	-	Ser	Gly	
CPV-2c^c^	-	-	Arg	Leu	-	Asn	-	Ala	-	Ile	-	Ala	Gly	Tyr	Asn	-	-	-	Glu	-	-	-	-	-	-	Ser	Gly	
702/00	Ser	-	-	-	-	-	-	-	-	-	-	-	-	-	-	-	-	-	-	-	-	-	Arg	-	-	-	-	FPV
713/01	-	-	-	-	-	-	-	-	-	-	-	-	-	-	-	-	-	-	-	-	-	-	-	-	-	-	-	FPV
671/02	-	-	-	-	Ser	-	-	-	-	-	-	-	-	-	-	-	-	-	-	-	-	-	-	-	-	-	-	FPV
1759/03	-	-	-	-	-	-	-	-	-	-	-	-	-	-	-	-	-	Val	-	-	-	-	-	-	-	-	-	FPV
1306/04	-	-	-	-	-	-	-	-	-	-	-	-	-	-	-	-	-	-	Ser	-	-	-	-	-	-	-	-	FPV
339/06	Ser	-	-	-	-	-	-	-	-	-	-	-	-	-	-	-	-	-	-	-	-	-	-	-	-	-	-	FPV
1034/09	-	-	-	-	-	-	-	-	-	-	-	-	-	-	-	-	-	-	-	-	-	-	-	-	Ser	-	-	FPV
1469/02_1	-	-	Arg	Leu	-	Asn	-	Ala	-	Ile	-	Ala	Gly	Tyr	Asn	-	-	-	Glu	-	-	-	-	Asn	-	Ser	Gly	CPV-2c
1469/02_2	-	-	Arg	Leu	-	Asn	-	Ala	-	Ile	-	Ala	Gly	Tyr	Asn	-	-	-	Glu	-	-	-	-	-	-	Ser	Gly	CPV-2c
1469/02_3	-	Asp	Arg	Leu	-	Asn	-	Ala	-	Ile	Asp	Ala	Gly	Tyr	Asn	-	-	-	Glu	-	-	-	-	-	-	Ser	Gly	CPV-2c
1469/02_4	-	-	Arg	Leu	-	Asn	-	Ala	-	Ile	-	Ala	Gly	Tyr	Asn	-	Val	-	Glu	-	-	-	-	-	-	Ser	Gly	CPV-2c
1469/02_8	-	-	Arg	Leu	-	Asn	Ser	Ala	-	Ile	-	Ala	Gly	Tyr	Asn	-	-	-	Glu	-	-	-	-	-	-	Ser	Gly	CPV-2c
1469/02_10	-	-	Arg	Leu	-	Asn	-	Ala	-	Ile	-	Ala	Gly	Tyr	Asn	Val	-	-	Glu	-	-	-	-	-	-	Ser	Gly	CPV-2c
998/08_1	-	-	-	-	-	-	-	-	-	-	-	-	-	-	-	-	-	-	-	-	-	-	-	-	-	-	-	FPV
998/08_6	-	-	Arg	Leu	-	Asn	-	Ala	-	Ile	-	Ala	Gly	Tyr	Asn	-	-	-	-	Ala	Pro	-	-	-	-	Ser	Gly	CPV-2a
998/08_9	-	-	-	-	-	-	-	-	-	-	-	-	-	-	-	-	-	-	-	-	-	Ser	-	-	-	-	-	FPV
998/08_15	-	-	Arg	Leu	-	Asn	-	Ala	Pro	Ile	-	Ala	Gly	Tyr	Asn	-	-	-	-	Ala	-	-	-	-	-	-	-	CPV-2a

In order to simplify the presentation of results, the identical sequences at the amino acid level have not been included in the table. The host-specific amino acids which distinguish the FPV and CPV strains are represented by grey dashes.

Deduced amino acid sequences of the VP2 gene were obtained from the GenBank:

^a ^Prototype FPV: reference strain FPV-b accession number: M38246

^b^Prototypes CPV: type 2: CPV-b (M38245), type 2a: CPV-15 (M24003), type 2b: CPV-39 (M74849)

^c^Reference strains: type 2a: CPV-677 (AF306445), type 2b: CPV-637 (AF306450), type 2c: CPV-695 (AF01519)

Sample 1469/02 showed unusually high genetic diversity generated within the infected host; six of the 10 (60%) mutations were non-synonymous. Coding changes located in residues 46 (Asn→Asp), 98 (Leu→Ser), 340 (Ala→Val), 373 (Asp→Val) and 511 (Asp→Asn) have not previously been reported while the mutation affecting position 282 (Asn→Asp) has already been reported [17].

The 998/08 sample also displayed very high genetic complexity; on the basis of the VP2 amino acid sequences, we were able to detect four main viral populations: clones 1, 3, 4, 7, 12 and 13 resemble the predominant FPV strains circulating in our area since these clones are identical to the majority of the FPV strains analyzed in this study while clone 9 is an FPV strain with unique characteristics, and is distinguishable from the other clones due to the presence of coding change VP2 Leu 498-to-Ser. Clones 6 and 20 are typical CPV-2a with a coding change located at position 482 (Leu →Pro), and finally clone 15, is a true intermediate between CPV and FPV, since analyzing the 10 important host-specific amino acids of FPV and CPV, showed that there were eight residues identical to CPV-2a and two amino acids identical to FPV (Table 2).

Summaries of sample sequence variability are presented in Table 3. The sequence data are analyzed separately on the basis of viral typing, grouping the sequences into three different subpopulations: FPV (all typical FPV strains), 1469/02 (all clones of sample 1469/02) and 998/08 (all clones of sample 998/08). The data are compared to samples showing analogous characteristics of genetic diversity and previously analyzed in our laboratory [8,18]. The sequence variability of the 998/08 sample was higher than the variability of the other sequence data analyzed, as was seen by the values of parameter π. A total of 34 polymorphic sites were found scattered within the sequence data of sample 998/08. The non-synonymous fraction was greater for sample 1469/02, indicating a clear prevalence of the number of non-synonymous mutations in this sample. In fact, in the clones of 1469/02, there were 6 non-synonymous mutations out of a total of 10 mutations.

**Table 3:** Summaries of sample sequence variability.

Sample	***S***^**b**^	***π***^**c**^	***SynDif***^**d**^	***NSynDif *(a)**^**e**^	***NSyn fraction***^***f***^
FPV (*n*^a ^= 22)	37	0.00551 (SE 0.00049)	31	6	0.16
1469/02 (*n *= 8)	10	0.00153 (SE 0.00018)	4	6	0.60
998/08 (*n *= 10)	34	0.00913 (SE 0.00266)	19	15	0.44
116/05 (*n *= 14)	19	0.00156 (SE 0.00036)	7	12	0.63
231/03 (n = 3)	6	0.00229 (SE 0.00108)	4	2	0.33

^a ^*n*, sample size

^b ^*S*, polymorphic sites

^c ^*π*, nucleotide diversity

^d ^*SynDif*: total number of synonymous differences

^e ^*NSynDif*: total number of non-synonymous differences

^f ^*NSyn fraction*: non-synonymous fraction

For samples 1469/02 and 998/08, the mutation frequency and the percentage of mutated viral clones were used as indicators of population diversity. The mutation frequency was of the order of one mutation every 7.2 × 10^-4 ^nt to 2 × 10^-3 ^nt (Table 4).

**Table 4:** Quasispecies variation in the viral populations detected within infected hosts.

Sample	% Mutated clones	Total mutations/bases sequenced	Mutation frequency
1469/02	100 (8/8)	10/13960	7.2 × 10^-4^
998/08	60 (6/10)	34/17450	2 × 10^-3^
116/05	57 (8/14)	19/24430	7.8 × 10^-4^
231/03	67 (2/3)	6/5235	1.1 × 10^-3^

To evaluate the selection pressure which acts on the VP2 gene, the ratio of synonymous (dS) and non-synonymous substitutions (dN) per site was calculated for the entire gene; the values of the dN/dS ratio were estimated as 0.27 for the entire sequence data set, 0.04 for the FPV dataset, 0.33 for sample 1469/02 and 0.18 for sample 998/08. A maximum likelihood approach was used for detecting the sites under selection in the viral clones. All methods implemented in the Datamonkey web interface were run and a comparative analysis integration was carried out. When the entire sequence data set was analyzed, the SLAC and FEL methods did not detect sites subject to positive selection but they found 8 and 32 sites under negative selections. The only REL analysis has detected 3 positively selected sites when the value of the Bayes factor was 100 (codons 13, 426, 564). The pressure analyses were carried out separately on the clone data sets, with the aim of investigating which selective forces acted during intra-host infection. All methods pointed out several sites under negative selection for samples 998/08 and 1469/02, and the REL method showed that all sites were under purifying selection for sample 1469/02. No potential recombination events were detected in the data set of the viral clones.

**Figure 1:**
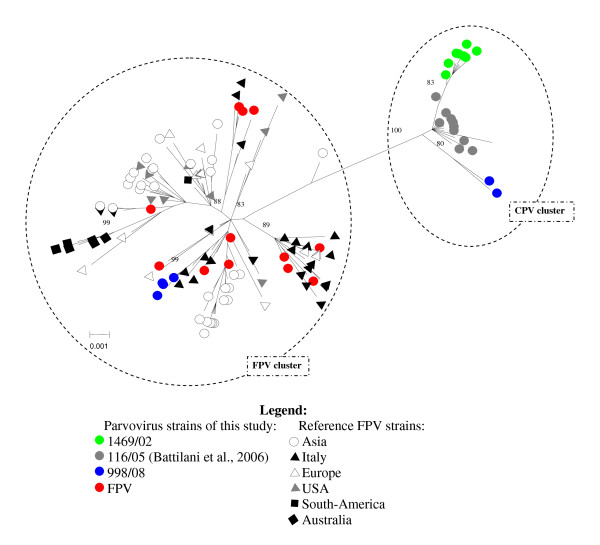
**Unrooted phylogenetic tree constructed with the nucleotide sequences of the VP2 gene of parvovirus strains**. Phylogenetic tree was constructed with the nucleotide sequences of the VP2 gene of parvovirus strains generated in this study and other FPV sequences obtained from the GenBank database. Horizontal branch lengths are drawn to scale (nucleotide substitutions per site), and bootstrap values above 70% are indicated on the respective branches. Identical VP2 sequences were not included in the analysis.

A phylogenetic tree was constructed from the VP2 nucleotide sequences obtained in this study and additional sequences were retrieved from the GenBank database. Unrooted tree showed two main clusters supported by elevated bootstrap values: FPV clade one and CPV clade one. The CPV cluster included all clones of sample 1469/02 and the CPV-2a clones of sample 998/08, with clone 15 of sample 998/08 forming a monophyletic branch. Inside the FPV cluster, a clear clustering on a temporal or geographical basis was not noted (Figure 1). Many FPV strains analyzed in this study clustered with Italian, European and Asian FPV strains. A recent FPV strain isolated from diarrhoeic monkeys [19] forms a separate lineage between the two clusters.

## Discussion

### FPV infections of cats

The present study explored the molecular characteristics of parvovirus strains circulating in cat populations and, although limited in the number of sequences analyzed, provides important new findings about the evolutionary dynamics of parvoviruses in cats and the host-virus relationships. Current results have demonstrated that the majority of feline panleukopenia outbreaks are caused by classic FPV strains and that FPV varies at a slow rate by random genetic drift, without a clear temporal or geographical distribution, as has already been reported in a recent study [6]. Considering all the nucleotide changes encountered in the VP2 sequences of FPV, synonymous substitutions predominated over non-synonymous substitutions, and the majority of FPV strains are identical at the amino acidic level. In spite of the low level of variability evidenced in the capsid gene, among the non-synonymous mutations of FPV sequences, one change involving an important epitope for CPV, residue 426, should be noted. Changes in residue 426 have modified the antigenic profile of CPV since it is located in the major antigenic region over the three-fold spike of the CPV capsid, and molecular epidemiological studies have demonstrated that complicated selection dynamics act on this residue [20]. Residue 426 has undergone two mutations since the emergence of CPV, first from asparagine to aspartate and, more recently, to glutamate, amino acid changes which constitute an important genetic marker for the new variants of CPV, and the only coding changes capable of distinguishing the CPV-2a strains from CPV-2b/2c strains [21]. The biological consequences of these mutations are not clear, although it has been observed that the new variants have acquired definite advantages over the original CPV-2. Mutations involving residue 426 have not previously been reported for FPV in the literature; therefore, the exact consequences of the change Asn-426 to Ser detected in FPV strain 1306/04 are not known but, analogously to CPV, it may be hypothesised that this change could affect the antigenic profile of FPV and confer an evolutive advantage to the virus. Further studies and investigations on other FPV strains detected from other parts of the world are needed to clarify whether this mutation is fixed in the FPV population as a consequence of the mechanisms of antigenic escape or further adaptation to the feline host.

### Multiple CPV infections of cats and evidence for coinfection

The diffusion of CPV in domestic cats and the pathogenic potential of CPV in causing infection or disease in cats have not been systematically investigated. Our report confirms that feline CPV infections are rare in Europe, since CPV is found only sporadically in feline biological samples [5].

A particularly significant finding in this study was the unusual genetic complexity reported for sample 1469/02, with 8 different viral populations belonging to type 2c of the CPV which coexisted in an infected cat, a phenomenon which resembles the quasispecies distribution found in RNA virus populations.

It is interesting to note that, in sample 1469/02, only the antigenic CPV variant 2c was detected, and this variant has already been reported in multiple infections of high genetic complexity. The genetic diversity displayed by sample 1469/02 was analogous to the high levels of sequence variation observed for a CPV sample (116/05) analyzed in our previous report, where a coinfection with two antigenically CPV variants 2a and 2c in a domestic cat was detected (Table 3) [8]; in that case, superinfection with many CPV variants was the source of such genetic diversity. In addition, we reported a co-infection in a dog with CPV variants 2a and 2c [18].

Experimental data and field observations reported a more severe clinical course and higher mortality rates associated with CPV-2c infection as well as an ability to infect and cause diseases in adult dogs, even if repeatedly vaccinated, suggesting that the Glu-426 mutation could provide a certain advantage in viral replication [22].

The unique antigenic pattern exhibited by CPV-2c could confer biological advantages on this variant, such as the possibility of escaping the adaptive immune system and the chance of increasing the complexity and diversity of the quasispecies. Diversity in viral quasispecies has been described as a mechanism for avoiding host resistance responses or a reservoir for maintaining variants with a selective advantage in other environments, and it has been correlated with the ability of infecting numerous hosts [23], having relevant biological implications for viral evolution and pathogenesis.

Furthermore, it is necessary to point out the genetic complexity observed in another sample (998/08), analyzed in this study. Sample 998/08 contained two distinct species of parvovirus, FPV and CPV type 2a, with the presence of one parvovirus variant, clone 15, that showed intermediate characteristics between FPV and CPV-2a. Clone 15 presented at positions 80, 93, 103 and 323, the same residues as typical CPV-2a, but residue 564 and 568 displayed amino acids Asn and Ala which are typical of FPV sequences. Since Datamonkey analysis excluded the fact that recombination events took place, it has been hypothesized that these coding changes are the result of de novo mutations arising during in vivo infection as an adaption of CPV into the new environment. Since cats constitute a relatively new host for CPV, the changes in positions 564 and 568 could increase viral fitness and be the result of virus adaptation on the new host species. Furthermore, the mutation frequency detected in sample 998/08 was of the order of 2 × 10^-3^, a value analogous to the RNA virus, and higher when compared to the annual substitution rate of the order of 10^4 ^to 10^5 ^nt. estimates of carnivore parvovirus. This result supports the importance of coinfection as a potential source of genetic diversity, confirming previous reports about the intrahost diversity of parvoviruses [9]. On the other hand, the higher genetic heterogeneity detected in sample 998/08 points out the predominant epidemiological role of cats as (a) carriers of carnivore parvovirus since it is susceptible to both viruses and (b) sources of new variants of parvoviruses [24]. Although coinfection with CPV and FPV would be a plausible event, to the author's knowledge, it has not previously been reported in the natural state. Our study demonstrated that CPV-FPV coinfection in cats has generated more variants for both species and, in particular, a CPV variant which is a true intermediate between the two species has been identified. A previous study demonstrated that CPV has emerged as a host range variant of FPV and this variant has acquired the ability to infect dogs through multistep changes in its capsid protein controlling critical molecular interaction with host cell receptor TfR binding [25]. This type of multistep adaptation has already been seen for other viruses, such as the influenza virus, and it is favoured when viable intermediate viruses can be selected [26]. The emergence of CPV has also involved the interspecies transmission between wild and domestic carnivores, a hypothesis confirmed by the detection of FPV-like strains with intermediate characteristics between CPV/FPV [27]. The report of clone 15 in sample 998/08 which contained FPV- and CPV-specific epitopes stresses the importance of this mechanism of multistep mutation in the production of new variants and in the emergence of new viruses.

In conclusion, this study provides new important results about the evolutionary dynamics of CPV infections in cats, showing that CPV has presumably started a new process of readaptation in the feline host and confirming the importance of viral host switching as a mechanism for the emergence of new viruses. The detection of mixed FPV/CPV infections in cats, associated with the report of the high genetic diversity of CPV-2 when it infects and replicates in cats, indicates the need to conduct additional studies in order to clarify the epidemiological role of cats in parvovirus infection, and it emphasizes the possible role of cats as a source of new variants of parvoviruses.

Furthermore, the introduction of CPV into the feline population raises some concerns about the efficacy of FPV-based vaccines in preventing CPV infection, and points out the necessity for intensifying surveillance of parvovirus infection in cats.

## List of Abbreviations

CPV: Canine Parvovirus; FPV: Feline Parvovirus; MEV: Mink Enteritis Virus; PCR: Polymerase Chain Reaction; SLAC: Single Likelihood Ancestor Containing; FEL: Fixed Effects Likelihood; REL: Random Effects Likelihood;

## Competing interests

The authors declare that they have no competing interests.

## Authors' contributions

MB: design of the study, carried out the molecular genetic studies, performed the statistical and phylogenetic analysis, and drafted the manuscript.

AB: carried out the molecular analysis of the biological specimens, participated to the drafting of the manuscript.

MU: carried out the molecular analysis of the biological specimens.

MG: performed clinical diagnosis, collected the samples and helped to draft the manuscript.

AS: participated to the statistical analysis and to the drafting of the manuscript.

SP: coordinated and participated to design of the study

All authors read and approved the final manuscript.
